# Urinary tract infections in the geriatric patients

**DOI:** 10.12669/pjms.341.14013

**Published:** 2018

**Authors:** Yesim Alpay, Nevil Aykin, Pinar Korkmaz, Hakki Mustafa Gulduren, Figen Cevik Caglan

**Affiliations:** 1 Yesim Alpay, M.D. Asst. Professor, Department of Infectious Disease and Clinical Microbiology, Balikesir University School of Medicine, Cagis, 10000, Balikesir, Turkey; 2 Nevil Aykin, M.D. Department of Infectious Diseases and Clinical Microbiology Tepebasi, Eskisehir Yunus Emre State Hospital, 2620190 Eskisehir, Turkey; 3 Pinar Korkmaz, M.D. Department of Infectious Disease and Clinical Microbiology, Dumlupinar University School of Medicine, Kutahya, Turkey; 4 Hakki Mustafa Gulduren, M.D., Department of Infectious Diseases and Clinical Microbiology Tepebasi, Eskisehir Yunus Emre State Hospital, 2620190 Eskisehir, Turkey; 5 Figen Cevik Caglan, M.D., Department of Infectious Diseases and Clinical Microbiology Tepebasi, Eskisehir Yunus Emre State Hospital, 2620190 Eskisehir, Turkey

**Keywords:** Antimicrobial Susceptibility, Complicating factors, Diagnosis, Geriatric, Microorganism, Urinary Tract Infection

## Abstract

**Objective:**

Urinary tract infections (UTI) are the second most common infection in geriatric population. This study investigated clinical findings, diagnostic approaches, complicating factors, prognosis, causative microorganisms and antimicrobial susceptibility in geriatric patients diagnosed with UTI.

**Methods:**

A total of 140 hospitalised patients with UTIs were evaluated within three years between January 2011-January 2015 at the Eskisehir Yunus Emre State Hospital. UTI diagnosed when there were systemic and urinary signs and symptoms and a positive dipstick test and urine culture result, leukocyte and CRP like serum parameters.

**Results:**

Among the studied patients, 41.4% had urological diseases, 20.7% had diabetes mellitus and 19.2% had neurological diseases. The most common symptoms and signs were fever, dysuria nausea/vomiting, general condition impairment, pyuria, haematuria. The laboratory values for CRP, ESR and leukocyte count were 84 mg/dL, 56 mm/s and 11.9 (10^3μL), with mean values being determined. Among patients having a urinary catheter (17.1%), 27.9% had a history of UTI, while 29.3% had been hospitalised. *Escherichia coli* and *Klebsiella pneumoniae* were the most commonly identified microorganisms. The mean duration for hospitalisation was 7.6 days, while a 5% mortality rate was observed over the course of the disease.

**Conclusion:**

Because of the potential for serious complications and mortality, elderly patients with urinary tract infection, should receive immediate empirical treatment based on anamnesis, clinical evaluation and urinalysis and should be re-examined using results from cultures and antibiograms upon follow-up.

## INTRODUCTION

Given the clinical impact of immunological ageing, the elderly suffer from increased sensitivity to bacterial and viral infections, with urinary tract infections (UTI) being the second most common infection in the geriatric population.^1^ Furthermore, urine and faecal incontinence, dehydration, impaired cognitive function and limited activity increase their susceptibility to infections.^2^,^3^

Diagnosis and treatment of UTIs are more complicated in elderly patients than in younger ones due to underlying factors, such as old age, diabetes mellitus (DM), spinal cord injuries, catheterisation and impaired general condition.^2^ Moreover, atypical symptoms can cause delays in the diagnosis.^3^

Factors facilitating the development of UTI include pelvic prolapse, cystocele, rectocele, bladder diverticulum, urinary reflux, incontinence, lack of perineal hygiene, vaginal atrophy, oestrogen deficiency in women and prostate diseases in men. Mental status changes, immunosuppression, DM, neurological diseases, invasive procedures, strictures and anatomical changes are among the main risk factors in the elderly.^3^,^4^

Given that the elderly do not present with typical findings for infectious diseases, it is important to note any changes in their previous status. As such, typical findings like UTI, dysuria, pollakuria and urgency may not always be seen in the elderly. However, incontinence, nausea, vomiting, abdominal pain, respiratory distress and changes in consciousness may accompany the diagnosis.^3^

Typical presentations of UTI and the difficulties in the management of infections once diagnosed can increase hospitalisation and mortality in the elderly. For this reason, physician suspicion is very important for the diagnosis of UTI in the elderly.^4^ Therefore, during clinical suspicion, geriatric patients undergo a pre-treatment urine culture and urinal dipstick test. Urinal dipstick test for the determination of predictive values and empirical antibiotic treatment, which are useful in decreasing morbidity and mortality.

This study investigated clinical findings, diagnostic approaches, complicating factors, prognosis, causative microorganisms and antimicrobial susceptibility in geriatric patients diagnosed with UTI. The aim was to emphasize some important points about urinary infections in the elderly and to discuss with the appropriate management of the disease and the reduced complications and mortality and prevents further increase in antimicrobial resistance caused by inappropriate treatment.

## METHODS

This study was conducted at the Eskişehir Yunus Emre State Hospital, Infectious Diseases and Clinical Microbiology Clinics. This study had been approved by the hospital's ethical committee with the following approval number and date: 56761182-900-1228/11.11.2014. A total of 140 hospitalised patients with UTIs were evaluated within three years between January 2011-January 2015 at the Eskisehir Yunus Emre State Hospital.

Demographic data, clinical symptoms, additional diseases, underlying urological pathologies, recurrent UTIs, traumas, hospital admissions, durations of hospitalisation, presence of urinary catheter and leucocyte, erythrocyte sedimentation rate (ESR) and C-reactive protein (CRP) values in the serum were recorded in detail during the first application.

The first urine specimens were sent to the laboratory for biochemical analysis, sterile medium-flow urine specimen analysis and microbiological examination. Results of the biochemical analysis were then recorded.

Medium-flow urine specimens taken for microbiological examination were inoculated with 5% sheep blood agar and eosin methylene blue agar and incubated at 37 °C for 24-48 h. A bacterial growth of 100,000 CFU/mL was considered significant. Isolation of more than one organism from a single specimen of urine interpreted as contamination.

Blood samples from at least two different veins were taken after the skin antisepsis. Blood culture bottles were incubated at 37°C in a Bactec 9120 (Becton Dickinson, USA) system. Isolated strains were evaluated using Gram staining. Identification and antibiograms were performed using the VITEK 2 ID-AST (BioMerieux, France) automated system. Antibiotic susceptibility was determined according to sensitivity and resistance values of the Clinical and Laboratory Standards Institute.^5^ Strains with intermediate susceptibility were considered resistant. Extended-Spectrum Beta-Lactamases (ESBL) production was determined using the double-disc synergy test. UTI diagnosed when there were systemic and urinary signs and symptoms and a positive dipstick test and urine culture result, leukocyte and CRP like serum parameters.

The SPSS Statistics software version 20.0 was used for statistical analysis. Descriptive statistical analyses and descriptive values of obtained data were presented as numbers with percentage frequencies. Student's t-test and Chi-square test were used to compare independent variables, with P <0.05 being considered statistically significant.

## RESULTS

During the five-year period, we evaluated 558 files of patients from the infectious disease clinic who were over the age of 65 years. A total of 140 hospitalised patients with UTIs were evaluated. The 140 patients included in this study had a mean age of 78.5 (65-98) years and a gender distribution of 53.6% female and 46.4% male. UTI rates according to age groups were 32.9%, 48.6% and 18.6% for those ageing 65-75, 75-85 and over 85 years.

Among the studied patients, 41.4% had urological diseases, 20.7% had DM, 19.2% had neurological diseases, 11.4% had chronic obstructive pulmonary disease, 7.1% had heart failure and 2.8% had other diseases. Underlying urological diseases are listed in Table-I.

**Table-I:** Underlying urological diseases.

Underlying urological diseases	Rate (%)
Urolithiasis	15
Benign prostatic hypertrophy	14.2
Tumour	7.1
Renal failure	3.5
Horseshoe kidney	1.4
Urethral stricture	0.7

Among patients having a urinary catheter (17.1%), 27.9% had a history of UTI, while 29.3% had been hospitalised. The mean duration for hospitalisation was 7.6 (1–20) days, while a 5% mortality rate was observed over the course of the disease.

The laboratory values for CRP, ESR and leukocyte count were 84 mg/dL, 56 mm/s and 11.9 (10^3μL), with mean values being determined. Clinical and laboratory features of the patients are given in Table-II.

**Table-II:** Clinical and laboratory features of the patients.

Assessed features	Number of patients (n)	Ratio (%)
Fever	108	77
Nausea	41	29
Vomiting	21	15
Abdominal pain	20	14
Polyuria	41	29
Dysuria	78	56
General condition impairment	48	34
Pyuria	131	94
Nitrite positivity	102	74
Haematuria	108	77
Secondary bacteremia	13	9
Antibiotic exchange rate	21	15

*Escherichia coli* and *Klebsiella pneumoniae* were the first and second most commonly identified microorganisms, with ESBL ratios of 49% and 66%, respectively. The distributions of causative microorganisms are listed in Table-III. Antimicrobial susceptibility of microorganism listed in Table-IV.

**Table-III:** Distributions of causative microorganisms.

Microorganism	Frequency (%)
Escherichia coli	52 (66)
Klebsiella pneumoniae	12 (15)
Pseudomonas aeruginosa	6 (8)
Enterococcus spp.	5 (6)
Acinetobacter baumannii	2 (3)
Staphylococcus aureus	1 (1)
Candida spp.	1 (1)
Contamination/no growth	61 (43.6)
Total	140 (100)

**Table-IV:** Antimicrobial susceptibility of microorganism.

Microorganisms Antimicrobials	E. coli (n:52)	K. pneumoniae (n:12)	P. aeruginosa (n:6)	A.baumannii (n:2)	Enterococcus spp (n:5)	S. aureus (n:1)	B.albicans (n:1)
Amoxicillin/clavulanic acid	20/52	4/12	-	-	-	-	-
Cefepime	16/52	3/12	3/6	1/2	-	-	-
Ceftazidime	15/52	3/12	3/6	2/2	-	-	-
Cefotaxime	10/52	3/12	2/6	1/2	-	-	-
Ceftriaxone	20/52	4/12	2/6	2/2	-	-	-
Amikacin	41/52	11/12	3/6	2/2	-	-	-
Piperacillin/tazobactam	42/52	12/12	3/6	1/2	-	-	-
Colistin	52/52	10/12	6/6	2/2	-	-	-
Cefoperazone/sulbactam	47/52	10/12	5/6	2/2	-	-	-
Tobramycin	26/52	6/12	4/6	2/2	-	-	-
Imipenem	51/52	12/12	3/6	1/2	3/5	1/1	-
Ciprofloxacin	18/52	4/12	3/6	2/2	3/5	1/1	-
Gentamicin	36/52	6/12	3/6	2/2	3/5	1/1	-
Trimethoprim/sulfamethoxazole	23/52	8/12	-	-	1/5	1/1	-
Linezolid	-	-	-	-	5/5	1/1	-
Vancomycin	-	-	-	-	5/5	1/1	-
Teicoplanin	-	-	-	-	5/5	1/1	-
Clindamycin	-	-	-	-	2/5	1/1	-
Oxacillin	-	-	-	-	-	1/1	-
Fluconazole	-	-	-	-	-	-	1/1
Itraconazole	-	-	-	-	-	-	1/1
Voriconazole	-	-	-	-	-	-	1/1
Caspofungin	-	-	-	-	-	-	1/1

## DISCUSSION

The prevalence and frequency of UTI in the geriatric population have been reported at variable rates due to differences in definitions. The frequency of UTI increases with older age in both sexes. In the current study, patients diagnosed with UTI were mostly between 75 and 85 years old and had a similar female to male ratio.^6^

UTIs are the most common type of infections (34%) requiring hospitalisation among the elderly in our country.^7^ In the current study, 25% of patients hospitalised due to geriatric infections had UTIs.

A history of UTI is one of the strongest predictive factors for its development in older individuals. Factors such as urinary incontinence, cystocele and DM increase the susceptibility to infections.^8^ Furthermore; elderly individuals have lower urination frequencies and fluid intake.

Age-related changes in immunity, multiple medical comorbidities, invasive procedures, prosthetic devices and short- and long-term urinary catheterisations increase the susceptibility to infections and the risk of hospitalisation.^9^ Among our patients, 27.9% had a history of UTI, 29.3% had been hospitalized and 17.1% had been catheterised. Moreover, 41% had additional urological diseases, such as urolithiasis and benign prostatic hypertrophy. Systemic diseases, such as DM and neurological diseases, were frequently observed. Similarly, Mahesh et al. had reported a strong association between UTI, urinary instrumentation and DM.^10^ In the geriatric population, medical symptoms may not be correctly identified due to neurological diseases, cognitive disorders and difficulties in cooperation.

One typical symptom is dysuria. Given that disorders of consciousness may cause difficulty in understanding the symptoms, care should be taken when recording patient anamnesis. Caregivers may observe the patient for signs of pain during urination. The presence of another clinical manifestation in addition to dysuria is 63% predictive for bacteriuria/pyuria.^11^

Despite fever being one of the most important indicators, 20%-30% of the cases may not experience it.^9^ In our study, fever was not seen in 23% of patients diagnosed with UTI. High fever is a clinical sign of a serious infection for which initiation of antibiotherapy becomes a necessity. It has also served as a criterion for the immediate initiation of empirical therapy in our patients.

Given that typical findings for infectious diseases are not readily observed in the geriatric population, changes in the patient's current condition need to be monitored. Typical findings for UTI, such as dysuria, polyuria and urgency, are sometimes obscured in the elderly. However, nausea, vomiting, abdominal pain, incontinence, respiratory distress and changes in consciousness may accompany the disease.^4^ Moreover, dysuria, mental status changes and changes in urine character (haematuria and changes in colour and odour) may accompany bacteriuria and pyuria.^12^ A study evaluating fever, dysuria, pollakuria, haematuria and abdominal pain in 194 geriatric patients with UTI showed that fever and dysuria were the most frequently observed symptoms, similar to our results.^10^

In our patients, fever and dysuria were predominant. In the general case of serious disorder one third was seen in a patient. Elevated leukocytosis and CRP values were observed at variable rates. Urinalysis revealed pyuria, nitrite positivity and haematuria in majority of the patients. Complicating factors and hospitalisation and UTI history were also evaluated. Prior to treatment, culture samples were obtained for microbiological examination, and empirical antibiotherapy was started depending on the findings and clinical assessments. Surveillance data, as well as patient and disease evaluations, were considered in determining the antibiotics of choice.

Although different criteria have been established, there is no clear consensus for the diagnosis of UTI in the geriatric population. In two studies involving older adults with UTI, Nicolle and colleagues suggested that the diagnosis be based on comprehensive clinical evaluation. New-onset urinary symptoms, the distinction of these symptoms from chronic symptoms, the presence of new findings and symptoms localised within the genitourinary tract and the possible exclusion of other diagnoses may indicate the presence of UTI. Clinical evaluations have emphasized that patients who often cannot adequately describe their symptoms are at a disadvantage during diagnosis.^13^ There is a guideline on the diagnosis of UTI that is based on clinical signs and symptoms and not primarily onlaboratoryfindings.^14^

Although urine culture is the gold standard for the diagnosis of UTI, it is expensive and time consuming. Given the need to obtain results within at least 48 hour, the first choice for clinicians in the initial patient evaluation was urinalysis using the dipstick test.^15^ Aside from being quick and inexpensive; dipstick tests do not require much experience.

Contaminant cultures are frequently done in the older population because of decreased self-care, poor hygiene, faecal contamination and cognitive disorders. They are also common for inappropriate urine specimens. Indeed, a high rate of contamination was present in our urine cultures. In fact, our study had a culture result that was determined to have high contamination.

UTI is diagnosed when there are systemic and urinary signs and symptoms and a positive dipstick test and urine culture result, leukocyte and CRP like serum parameters, in our study. UTI diagnosis in culture-negative patients were not excluded if it is supported by other parameters. One of the leading causes of culture negativity was the use of antibiotics during the application of some of the patients. Mostly, parenteral treatment is necessary because of general condition disorder, nausea, vomiting, oral intake disorder. In the presence of pre-culture antibiotic therapy, non-culturable organisms and some conditions like pyogenic abscess of kidney and perinephric tissues, prostatitis, pyelonephritis or obstructed pyonephrosis, urine may be sterile. Moreover, asymptomatic bacteriuria is common in the geriatric group and clinical findings and the presence of pyuria are important for treatment. For all these reasons, diagnostic approaches other than urine culture are strongly considered.

Dipstick tests, which correlate with clinic status, can serve as a first level screening in outpatient clinics/primary health care centres.^10^ The negative predictive value was high for a negative nitrite test. In a recent meta-analysis, a negative urinary dipstick test was reported to be accurate and useful in excluding UTIs.^16^ Causative bacterial agents of UTIs reduce nitrate to nitrite. Excluding Enterococci, Candidae and Streptococci, almost all Enterobacteriaceae and non-fermenting bacteria reduce nitrate.^15^

Causative agents of UTI in the elderly are often Gram-negative bacteria, most commonly *E. coli*.^17^ Gram-negative bacteria are frequently observed due to faecal contamination and decreased perineal hygiene. The nitrite test has been reported to have the highest sensitivity and accuracy in the elderly population.^18^

In our study, *E. coli* (66%) and *K. pneumoniae* (15%) were the most common causative agents, as well as the most commonly reported causative agents in different studies across our country.^19^,^20^ In their study, Mahes and colleagues showed that *E. coli* (77.1%) was the most common among the strains, with 56.2% of these strains producing ESBL (+).^10^ ESBL ratios in *E. coli* and *Klebsiella* strains have been increasing. Similarly, the present study obtained ESBL ratios of 49% and 66% for *E. coli* and *Klebsiella*, respectively. ESBL-producing microorganisms are resistant to many antibiotics and are associated with morbidity and mortality due to treatment failure.^10^ In particular, fluoroquinolone resistance has been more prevalent in ESBL-producing strains.^21^ Recently, ciprofloxacin resistance has been increasing, with our study showing ciprofloxacin resistance rates of 65.4% and 66.6% in *E. coli* and *K. pneumoniae*, respectively. In a study performed in our country, high rates of ciprofloxacin, gentamicin, ampicillin and cefazolin resistance were reported^19^, with the present study observing similar patterns of resistance. The most effective antibiotics against *E. coli* and *K. pneumoniae* strains were determined to be imipenem, amikacin, cefoperazone-sulbactam and piperacillin-tazobactam. Linezolid, vancomycin and teicoplanin were most effective against Gram-positive microorganisms.

UTIs are considered to be the most common cause of bacteraemia among geriatric individuals, leading to high rates of hospitalisations.^22^ A study evaluating 100 bacteraemic episodes in the elderly population revealed that the genitourinary system was the most common source of bacteraemia.^23^ Although patients with secondary bacteraemia were detected in the current study, their prognosis remained good with immediate and appropriate empirical treatment. A 5% mortality rate was observed throughout the course of the disease. Another study evaluating bacteraemic UTIs obtaineda16.1% mortality rate, which was higher than that observed in the present study.^23^

Inadequate UTI treatment in elderly patients was often associated with worsening clinical outcomes. Moreover, 40%–75% of clinician-prescribed antibiotics for UTIs were not appropriate.^24^,^25^ Hence, given the predominance of Gram-negative bacteria, empirical antimicrobial treatment may have a positive effect on prognosis.^8^ Antimicrobial resistance constitutes an important problem in both community-based and hospital-acquired infections. Thus, surveillance data, such as national and regional antibiotic resistance rates, rates of resistant microorganisms and ESBL positivity, could facilitate good treatment responses using our empirical antimicrobial approaches.

Our results emphasise the difficulty of UTI diagnosis in geriatric patients where in atypical symptoms may be present instead of typical ones. Because of the potential for serious complications and mortality, elderly patients should receive immediate empirical treatment based on anamnesis, clinical evaluation and urinalysis and should be re-examined using results from cultures and antibiograms upon follow-up. Older age can lead to rapid disease progression, with patients becoming more susceptible to complications. Complications of bacteraemia and urosepsis may result in mortality. Therefore, broad-spectrum antimicrobials should be selected for empirical antibiotic therapy, which should be narrowed down when necessary. Given the increase in antimicrobial resistance in recent years, knowledge and consideration of surveillance data have become imperative in making treatment decisions. Thus, improved treatment response leads to reduced complications and mortality and prevents further increase in antimicrobial resistance caused by inappropriate treatment.

### Authors' Contribution

**YA and NA:** Conception

**YA, NA, PK, HMG and FCC:** Data Collection.

**YA, NA, PK, HMG and FCC:** Analysis and interpretation of data.

**YA, HMG and FCC:** Literature Review.

**YA, NA and PK:** Drafting of manuscript and Critical revision.

All authors have contributed in finalizing the manuscript.
